# The current state of eukaryotic DNA base damage and repair

**DOI:** 10.1093/nar/gkv1136

**Published:** 2015-10-30

**Authors:** Nicholas C. Bauer, Anita H. Corbett, Paul W. Doetsch

**Affiliations:** 1 Department of Biochemistry, Emory University School of Medicine, Atlanta, GA 30322, USA; 2 Graduate Program in Biochemistry, Cell, and Developmental Biology, Emory University School of Medicine, Atlanta, GA 30322, USA; 3 Winship Cancer Institute, Emory University School of Medicine, Atlanta, GA 30322, USA; 4 Department of Radiation Oncology, Emory University School of Medicine, Atlanta, GA 30322, USA; 5 Department of Hematology and Medical Oncology, Emory University School of Medicine, Atlanta, GA 30322, USA

## Abstract

DNA damage is a natural hazard of life. The most common DNA lesions are base, sugar, and single-strand break damage resulting from oxidation, alkylation, deamination, and spontaneous hydrolysis. If left unrepaired, such lesions can become fixed in the genome as permanent mutations. Thus, evolution has led to the creation of several highly conserved, partially redundant pathways to repair or mitigate the effects of DNA base damage. The biochemical mechanisms of these pathways have been well characterized and the impact of this work was recently highlighted by the selection of Tomas Lindahl, Aziz Sancar and Paul Modrich as the recipients of the 2015 Nobel Prize in Chemistry for their seminal work in defining DNA repair pathways. However, how these repair pathways are regulated and interconnected is still being elucidated. This review focuses on the classical base excision repair and strand incision pathways in eukaryotes, considering both *Saccharomyces cerevisiae* and humans, and extends to some important questions and challenges facing the field of DNA base damage repair.

## INTRODUCTION

Few systems are as crucial to sustaining life as DNA repair. The genomes in the cells of all organisms are under constant bombardment by genotoxic stresses, both exogenous (e.g. ultraviolet and ionizing radiation, chemical combustion products) and endogenous (e.g. reactive oxygen species, nucleases). These agents can modify the chemical structure of DNA in ways that produce mutations in transcribed RNA and replicated DNA, alter the ability of regulatory elements to be recognized by DNA binding proteins, and lead to cell death by blocking transcription and replication (1,2). Lesions can occur to most parts of the DNA structure, ranging from minor and major chemical modifications, to single-strand breaks and gaps, to full double-strand breaks. Chemical modifications are the most common lesions (3) while double-strand breaks are the most lethal (4). In eukaryotes, these lesions may occur in both nuclear and organellar (mitochondria, chloroplasts) genomes. The constellation of extraordinarily well conserved DNA repair pathways is responsible for removing these lesions and/or mitigating their effects. Properly repairing the common base lesions is important not only to abrogate their immediate impacts, but also to prevent their conversion into more deleterious strand breaks. These repair and tolerance mechanisms have important implications for human health and disease, especially oncogenesis and degenerative disorders associated with aging (5–7). This article reviews the most common and mutagenic classes of lesions and the pathways responsible for their repair, with a focus on the members of the classical base excision repair (BER) pathway first delineated by Tomas Lindahl (8), and recent work that has expanded the impact and roles of this pathway. Finally, the review concludes with a discussion of some current questions and challenges facing the field of DNA base damage repair.

## BASE, SUGAR AND SINGLE-STRAND BREAK LESIONS

**Figure 1.**
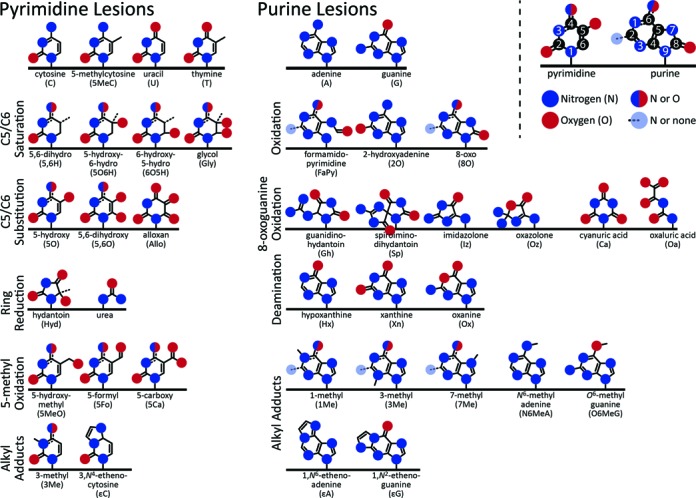
Common base lesions. The basic chemical structures of the common base lesions that occur in DNA grouped by type. The basic nucleotides are shown at the top, and lesions are displayed to indicate the same modification occurring to multiple bases. Hydrogens are omitted. IUPAC numbering for pyrimidines and purines is illustrated at the top right.

DNA base lesions, which are chemical modifications to the base of a nucleotide, are the most common type of genomic damage: an estimated 120,000 base lesions occur in the 6.5 Gbp nuclear genome of human liver cells per day (3). Base lesions may be accompanied more rarely by sugar modifications and single-strand breaks. These lesions can have serious consequences for numerous cellular processes, resulting in genomic mutagenesis (i), transcriptional mutagenesis (ii), and disruption of regulatory DNA elements (1). There are four major classes of base lesions: oxidation, deamination, alkylation, and hydrolysis. These lesions are detailed below and are illustrated in Figure 1.

### Oxidation

Reactive oxygen species (ROS; e.g. hydrogen peroxide, hydroxyl radical, superoxide anion) are critical components of normal signaling pathways (9) yet they are also a significant source of base damage (9). As a result, these chemical species are very carefully regulated, deliberately produced by oxidases and removed by scavengers. ROS can also originate from the environment, both directly and as an aftereffect of the reactions of antioxidants and xenobiotics (10). In addition, hydroxyl radicals can be produced by ultraviolet (UV) radiation in the UV-A band (315–400 nm) (11). Radiolysis of water by ionizing radiation also produces ROS in addition to reactive free protons and electrons which can produce similar sets of base lesions (12). Thus, there are numerous sources of ROS that can lead to formation of oxidative lesions.

Oxidative attack on pyrimidines [cytosine (C), 5-methylcytosine (5MeC), thymine (T)] can result in the saturation of the double bond between pyrimidine carbons 5 and 6 to form hydrate (5-hydroxy-6-hydro, 6-hydroxy-5-hydro) and glycol derivatives. The 5,6-dihydro derivatives are formed exclusively by the free protons and/or electrons generated by ionizing radiation. Carbon 5/6 hydrogens can be substituted to form 5-hydroxy and 5,6-hydroxy derivatives. C and 5MeC saturated lesions are especially prone to deamination, leading to the uracil (U) and T forms of the lesion, respectively. Uracil glycol rapidly decomposes to 5-hydroxyuracil (13–15). An exception to this instability is 6-hydroxy-5-hydrocytosine (13). Further oxidation of C (and U) can also lead to alloxan and then 5-hydroxyhydantoin while further oxidation of T results in 5-hydroxy-5-methylhydantoin. The 5-methyl group of 5MeC or T can be oxidized to 5-hydroxymethyl, 5-formyl, and 5-carboxy C and U derivatives, respectively. The oxidation of the 5-methyl group can be induced enzymatically as part of an active demethylation pathway in eukaryotes (16,17). Finally, any pyrimidine can be further oxidized to urea. Pyrimidine radical reaction mechanisms and products have been reviewed recently (12,18).

Oxidation of purines [adenine (A), guanine (G)] can produce ring-opened formamidopyrimidine derivatives, 8-oxo derivatives, and 2-hydroxyadenine. 8-Oxoguanine can undergo extensive further oxidation to the more mutagenic lesions, guanidinohydantoin, spiroiminohydantoin, imidazolone, oxazolone, cyanuric acid, oxaluric acid and urea. Purine oxidative reaction mechanisms and products are reviewed in (12,18,19).

Deoxyribose in DNA is also vulnerable to oxidation, which usually results in base hydrolysis to form an abasic (apyrimidinic/apurinic, or AP) site and/or a strand break in addition to the modified sugar. Deoxyribose oxidation can also result in a crosslink between the 5′ carbon of the sugar and carbon 8 of an attached purine, forming a cyclic nucleotide (12). There is a report from 1992 of oxidative conversion of deoxyribose to ribose *in vivo* (20), but no subsequent validation has been reported. An abasic site may also spontaneously form an *O*-glycosidic bond with an alcohol (21).

### Deamination

Deamination is the replacement of a nitrogen atom with an oxygen atom, primarily of exocyclic amines. Both C and 5MeC have an exocyclic amine on carbon 4. Deamination of these bases by a basic molecule produces U or T, respectively (22); deamination of 5MeC to T within the important regulatory CpG repeats likely makes up a large number of the observed mutations in cancer genomes (23). Purine deamination is mediated largely by the signaling radical nitric oxide. Deamination of the exocyclic amine at carbon 6 of A produces hypoxanthine (inosine). Deamination of the exocyclic amine at carbon 2 of G produces xanthine. Uniquely, the internal nitrogen 1 of G can be replaced by oxygen, producing oxanine (24).

### Alkylation

Alkylation is one of the less common types of base lesions, but they are often the most mutagenic (25). Methyl groups can be added to any available amine on both pyrimidines and purines as well as to the carbon 6 oxygen or nitrogen of G or A (26). Lipid peroxidation products can also react with an exocyclic amine and an adjacent internal amine in C, A or G to produce exocyclic etheno adducts or other adducts important for colon carcinogenesis including 2-propanoguanine (27,28). Xenobiotic metabolites (resulting from combustion products such as benzo(a)pyrene and other aromatic polyphenols) are highly reactive and thus able to form bulky base adducts (29,30).

### Hydrolysis

Hydrolysis of the *N*-glycosidic bond to generate an abasic site may occur spontaneously, via the action of a DNA *N*-glycosylase, or as part of a radical reaction mechanism (12). Hydrolysis of the sugar-phosphate backbone can also occur through a radical reaction mechanism, as discussed in Oxidation. The abasic site aldehyde is reactive and may progress to an interstrand crosslink to a purine on the opposing strand (31,32).

### Incorporation of damaged nucleotides

While most base damage is introduced into DNA directly, another source of lesions is from the incorporation of damaged bases from the nucleotide pool, particularly U and 8-oxoguanine. Recent studies demonstrated that misincorporation of ribonucleotides in DNA is a relatively common event, at a frequency of ∼4 for every 10^4^ nucleotides inserted in *S. cerevisiae* (33).

## BASE DAMAGE REPAIR PATHWAYS

**Figure 2.**
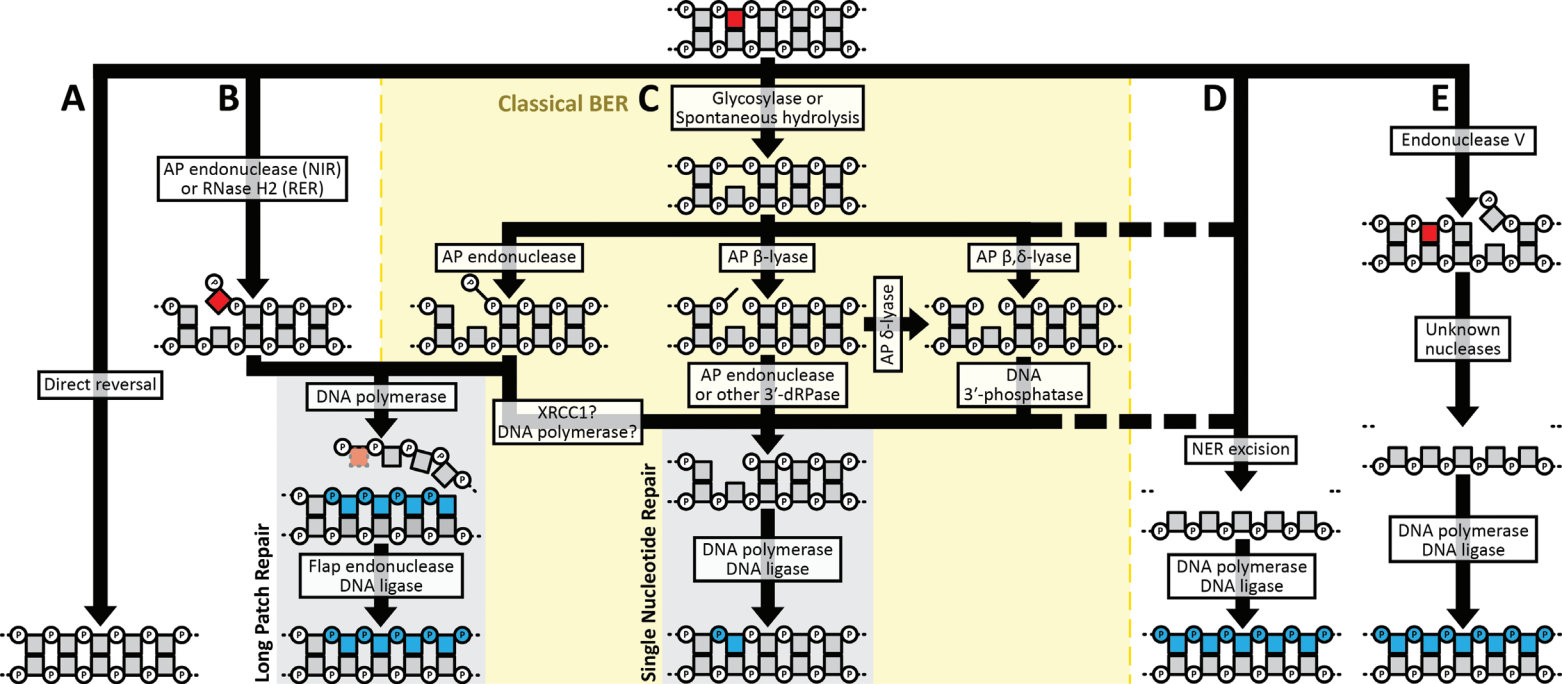
Base, sugar, and single-strand break lesion repair pathways. Base lesions can be processed by: (**A**) direct reversal; (**B**) nucleotide incision repair (NIR), ribonucleotide excision repair (RER); (**C**) classical base excision repair (BER); (**D**) nucleotide excision repair (NER) or (**E**) endonuclease V***–***mediated excision repair. Within the BER pathway (C), multiple semi-redundant pathways are available for processing abasic sites. Pathway choice depends on the lesion, and some lesions can be acted on by multiple pathways. Squares (nucleosides) and connected circles (phosphates) represent a section of a DNA molecule, with the base lesion indicated in red. *De novo* synthesized bases are shown in blue. The enzyme activity responsible for each step is noted. The dashed segments indicate a fallback pathway. AP = apurinic/apyrimidinic site; dRP = deoxyribose phosphate.

DNA base lesions pose a challenge for cells on an ongoing basis. The interconnected and overlapping set of pathways collectively known as DNA repair is responsible for either reverting the lesions back to the original form (repair) or otherwise limiting the potential impact of the lesion on cellular function (tolerance). Underscoring their critical role, these pathways are very highly conserved throughout all domains of life, and deleterious mutations in base damage repair genes are associated with neurodegeneration and cancer; for example, inherited mutations in the genes encoding the NTHL1 and MUTYH glycosylases have been linked to colorectal cancer (34–36). Typically, lesions can be repaired through multiple distinct, partially redundant pathways (37,38). The human and budding yeast *Saccharomyces cerevisiae* pathways are schematized in Figure 2 and reviewed in the following sections.

### Direct lesion reversal

Certain lesions can be directly removed from a base while leaving the DNA helix intact (Figure 2A). Many alkyl adducts can be removed by the AlkB family of dioxygenases (Human: ALKBH2, ALKBH3; *S. cerevisiae*: not present). ALKBH2/3 removes alkyl groups (39–41) and exocyclic adducts (42–44) at the nitrogen 1 position of purines and the nitrogen 3 position of pyrimidines. The most highly mutagenic alkylated base, *O*^6^-methylguanine, is reversed by *O*^6^-methylguanine methyltransferase (human: MGMT; *S. cerevisiae*: Mgt1), a ‘suicide protein’ which irreversibly transfers the errant methyl group onto itself and is subsequently degraded (45). Photolyases (human: not present; *S. cerevisiae*: Phr1) are light-dependent enzymes that directly reverse UV-induced pyrimidine dimers (46). Photolyases were lost early in the largely nocturnal mammalian lineage (47). While direct reversal efficiently restores DNA to its original pristine form, the majority of DNA damage is repaired through more general pathways that have the capacity to recognize and repair a spectrum of lesions.

### Base excision and strand incision repair

**Figure 3.**
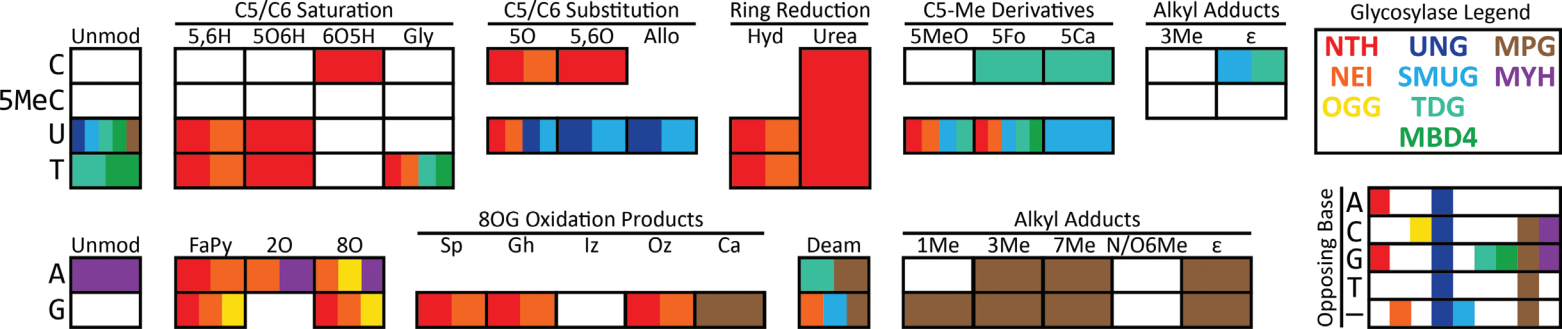
Substrate specificities of base excision repair glycosylases. Each square represents a DNA base lesion, with the row indicating the original base and the column indicating the particular lesion, grouped by shared features. ‘Unmod’ = unmodified. Refer to Figure 1 for lesion abbreviations. Coloration within each box indicates the glycosylase families that recognize the lesion and excise it from DNA, according to the legend at the top-right. The matrix at the bottom-right illustrates the specificity of the glycosylase families with respect to the base opposite from a lesion; — indicates single-stranded DNA. Note that this diagram does not account for differences in enzyme kinetics between or within families. Example: OGG (yellow) excises guanine-derived formamidopyrimidine, 8-oxoadenine, and 8-oxoguanine when these lesions are opposite to cytosine.

The base excision repair (BER) pathway efficiently corrects most non-bulky DNA base lesions that are not addressable by direct reversal. Thus, BER is responsible for repairing the vast majority of lesions that occur in DNA, and the pathway is active in both nuclei and mitochondria. BER is initiated by the recognition and hydrolysis of the damaged base by a DNA *N*-glycosylase, leaving an abasic site (48). The glycosylases are reviewed in depth below and their specific substrates are summarized in Figure 3. The BER pathway is illustrated in Figure 2C.

Abasic sites can be processed by one of two subpathways. The first subpathway, called single-nucleotide BER (SN-BER; also termed ‘short patch’) is initiated by an AP lyase. The AP lyase cleaves the DNA backbone on the 3′ side of the abasic site by β-elimination (AP β-lyase), which leaves a 3′-deoxyribose phosphate and 5′-phosphate, or by β,δ-elimination (AP β,δ-lyase), which excises the deoxyribose and leaves 3′- and 5′-phosphates (49). Both of these products block DNA polymerase activity. Removal of the 3′-deoxyribose phosphate (dRP) can be catalyzed by as little as a basic tripeptide, but is typically carried out by an AP endonuclease (49). Removal of the 3′-phosphate is catalyzed by the DNA 5′-kinase/3′-phosphatase, polynucleotide kinase/phosphatase (human: PNKP; *S. cerevisiae*: Tpp1) (50,51). The sequential action of an AP δ-lyase and DNA 3′-phosphatase can also process 3′-dRP as an alternative to AP endonuclease (52,53). The result is a clean 1-nucleotide gap, which is then directly filled by a DNA polymerase and sealed by a DNA ligase (54). The second subpathway, called long patch BER (LP-BER), is initiated by an AP endonuclease (human: APEX1, APEX2; *S. cerevisiae*: Apn1, Apn2), which cleaves the DNA backbone on the 5′ side of the abasic site (49). This incision leaves a 3′-hydroxyl group that can directly serve as a substrate for DNA polymerase β (human: POLB, *S. cerevisiae*: POL4). The polymerase fills the removed base and several bases downstream, displacing the strand on the 3′ side of the cut site (55). This displaced strand, which is terminated by the 5′-dRP, is removed by the flap endonuclease (human: FEN1; *S. cerevisiae*: Rad27) at its base and a DNA ligase seals the nick, leaving a fully repaired segment of DNA (48). An AP endonuclease–cleaved abasic site can also be directed into SN-BER through the 5′-dRPase activity of polymerase β (56). The mechanism controlling the switch between subpathways is unclear though it has been proposed that the human BER scaffold protein XRCC1 plays a role (57,58). Cellular ATP concentration, cell cycle phase, and chemistry of the 5′ terminus may also influence subpathway choice (59,60). The AP lyase activities, which promote SN-BER, are associated with the subset of glycosylases that recognize oxidative lesions (49,61–63). This subpathway may be favored for oxidative lesions which occur in clusters (54). Attempting LP-BER in such a cluster could result in polymerase stalling, mutagenesis, or converting the original simple base lesion into a more serious double-strand break (64,65).

Other processes produce lesions that may enter the BER pathway as intermediates. Bases can spontaneously hydrolyze from the DNA backbone to generate abasic sites with certain base lesions more prone to hydrolysis than other bases. Oxidative attack on deoxyribose can cause base hydrolysis and sugar damage (12), which are effectively processed by the coordinated actions of AP endonuclease, AP lyases and 3′-DNA phosphatases (66,67). Complex single-strand breaks can occur through the action of abortive topoisomerase or DNA ligase activity [via adenosine 5′-monophosphate (AMP)] adjacent to a lesion, which become trapped by covalent linkage to an end-group phosphate. Tyrosyl–DNA phosphodiesterases and aprataxin deadenylase, respectively, are able to reverse these polymerase-blocking groups (68,69). Resolution of these structures allows BER to complete repair. These end-trimming pathways were reviewed in depth recently (70).

Classical BER has been joined by two recently discovered strand incision repair pathways that feed into LP-BER: (i) nucleotide incision repair (NIR) and; (ii) ribonucleotide excision repair (RER) (Figure 2B). NIR is initiated by AP endonuclease, which is able to cleave the DNA backbone 5′ to pyrimidine lesions (71–77). As AP endonucleases are highly abundant, NIR can constitute the major repair activity against a subset of pyrimidine base lesions (74). RER is initiated by the RNase H2 complex (human: RNASEH2A-RNASEH2B-RNASEH2C; *S. cerevisiae*: Rnh202-Rnh203), which incises the strand 5′ to the misincorporated ribonucleotide (78). RNase H1 (human: RNASEH1; *S. cerevisiae*: Rnh1) has recently been shown to also incise 3′ to the ribonucleotide, producing a single-nucleotide gap (79), leaving open the possibility of feeding into SN-BER for completion of repair. Advances in RER and other repair pathways for ribonucleotides have been reviewed in depth (80).

The recent broadening of the range of lesions processed through BER, as well as the discovery of strand incision as a major alternate first step in the repair of many classic BER substrates, suggests that the term ‘base excision repair’ is no longer appropriate. Perhaps a more suitable term is ‘base excision and strand incision repair’ (BESIR), which more closely reflects the range of activities of the pathway components. The enzymes most critical for the initiation and processing of lesions in BESIR, the glycosylases, AP endonucleases, end-trimming enzymes, polymerases, and ligases are reviewed in the following sections.

#### Alkylpurine–DNA glycosylases

The alkylpurine–DNA glycosylases (human: MPG; S. cerevisiae: Mag1) are responsible for excising methylated purines (especially 3-methyladenine) (81–86), purine exocyclic adducts (e.g. 1,*N*^6^-ethenoadenine) (87,88), deaminated purines (e.g. hypoxanthine, xanthine, oxanine) (89,90), the 8-oxoguanine oxidation product cyanuric acid (91), uracil (86) and *O*-glycosidic additions to deoxyribose (21). Many of these substrates can be excised from both double-stranded and single-stranded DNA (86,90). Mag1 can also remove A mispaired with C (87). Intriguingly, MPG is able to catalyze the reverse reaction, forming an *N*-glycosidic bond with a free base, potentially allowing the correctly-paired base to be directly swapped (92). The biological relevance of this activity would be dependent on the relative concentrations of free bases in the nucleus, and it could be counterproductive if an incorrect base were inserted which was not excisable by MPG.

These glycosylases do not possess lyase activity and their resulting abasic sites can be processed both by SN- and LP-BESIR subpathways (63). Mag1 expression is inducible by the DNA damage checkpoint pathway (93–97) while MPG transcript levels are cell-cycle regulated (98). MPG has been identified both in the nucleus and mitochondria (99) while Mag1 is restricted to nuclei (100). MPG activity is enhanced by XRCC1 and a component of nucleotide excision repair, HR23 (101,102). MPG can bind to PCNA along with APEX1. APEX1 may stimulate MPG turnover by displacing MPG from the abasic site products, though the evidence to support this mechanism is mixed (103–105). MPG can also form a dimer with methylcytosine binding domain protein 1 (MBD1), which sequesters MPG at methylated CpG promoters. Upon alkylative attack on G, MBD1 dissociates from both MPG and the DNA allowing MPG to redistribute throughout the genome (85). This mechanism may provide an alkylation-responsive reservoir for rapid mobilization to repair alkylation damage.

#### Endonuclease III-like glycosylases

The endonuclease III-like *N*-glycosylase family (human: NTHL1; *S. cerevisiae*: Ntg1, Ntg2) is responsible for repairing a wide array of oxidative lesions in double-stranded DNA, primarily oxidized pyrimidines (e.g. 5-hydroxycytosine, cytosine hydrates, thymine glycol) (61,106–116), ring-fragmented purines (108,117–119), and 8-oxoguanine opposite a purine (120,121). There are several differences between these proteins with respect to substrate specificity. For example, Ntg1 and NTHL1 do not process 5-hydroxycytosine as efficiently as Ntg2 processes this lesion (74,106). Ntg1, however, is better at processing cytosine hydrates than is Ntg2. Ntg2 and NTHL1 can excise certain 8-oxoguanine oxidation products while Ntg1 does not (91,119,122). Ntg1 can process dihydrothymine while Ntg2 cannot (114). This family of enzymes possesses AP β-lyase activity which directs lesions into the SN-BESIR subpathway described above through a coordinated reaction mechanism, but this family can also contribute to the processing of abasic sites generated spontaneously or by monofunctional glycosylases (55,62,123,124).

NTHL1 activity on its own is over 100 times slower than is the activity of the bacterial counterpart, endonuclease III (125), and the rate-limiting step is release from the lyase-cleaved abasic site (126). APEX1 enhances release from that product (112). This mechanism may protect the toxic strand-break intermediate in a ‘passing the baton’ or ‘handoff’ substrate channeling mechanism (127). Other binding partners (e.g. XPG, YB-1, XRCC1) enhance the activity of NTHL1 though the precise mechanism behind the enhancement is unknown (102,112,125,128).

Ntg1 and Ntg2 are the result of a genome duplication in the evolutionary history of *S. cerevisiae* (129,130), and they have segregated certain characteristics that the single original enzyme possessed. NTHL1 and Ntg1 localize to both the nucleus and mitochondria and are involved in repairing both genomes while Ntg2 is strictly nuclear (106,114,131–136). On the other hand, NTHL1 and Ntg2 both contain a conserved iron-sulfur center but this feature is absent from Ntg1 (61,108,137). This iron-sulfur center is redox-active *in vivo* and has been hypothesized to be involved in DNA damage sensing. In brief, electrons can be transported over long distances through the π-orbitals of the DNA base pair stack between bound redox-active proteins containing iron-sulfur centers, a process called DNA-mediated charge transport. Reduction of the iron-sulfur center allows dissociation of the repair enzyme from the DNA. DNA lesions can disrupt the base stack, retarding charge transport. This condition causes the repair protein to remain bound to the DNA and slide along the helix until it encounters the lesion (138–141). Little is known about the transcriptional regulation of this family of proteins but NTHL1 is upregulated in S phase (118) and the Ntg1 promoter has a conserved (within yeast) promoter element necessary for oxidative stress induction (142).

#### Endonuclease VIII-like glycosylases

The endonuclease VIII-like *N*-glycosylase family (Human: NEIL1, NEIL2, NEIL3; *S. cerevisiae*: not present) is responsible for repairing a wide array of oxidative lesions primarily in single-stranded DNA. Most of the substrates of this enzyme family overlap with those of NTHL1 (74,115,117,119,143–156). NEIL1 and NEIL3 can also process 8-oxoguanine oxidation products in telomere-associated quadruplex DNA (157,158), NEIL3 can process thymine glycol in the same structures and is additionally able to process lesions near strand breaks which are refractory to NTHL1 and the 8-oxoguanine DNA glycosylase, OGG1 (159,160). NEIL1 can excise the internally deaminated G, oxanine (161). NEILs are also involved in repair of some interstrand crosslinks (162).

This family of enzymes possesses AP lyase activity. In contrast to NTHL1, NEIL1 and NEIL2 are AP β,δ-lyases (146,147) while NEIL3 employs the typical β-elimination mechanism (119). The β,δ mechanism directs NEIL1 or NEIL2-excised lesions into the SN subpathway, but there is also evidence that these lesions can undergo LP repair (163). NEIL1 or NEIL2, together with PNK, can provide backup dRPase activity to clean up after other AP β-lyases (53).

NEILs provide specialized repair activities in the cell. NEIL1 participates in pre-replication repair, in which NEIL1 recognizes lesions within the single-stranded region of the replication fork prior to being read by the DNA polymerase. NEIL1 excises the base and creates a strand break, forcing the polymerase to stall and backtrack so that the lesion can be repaired (164,165). NEIL2 associates with RNA polymerase II and CSB to allow repair of lesions encountered as transcription is progressing (143,157,166). There is some evidence from in vitro activity assays, immunoprecipitation, and HEK293 cell culture experiments that NEIL1 and NEIL2 can compensate for one another, but this ability appears to be limited (165,167,168).

NEIL1 is strongly upregulated during S phase (146) as well as under oxidative stress (169). In contrast, expression levels of NEIL2 are constant throughout the cell cycle (147) but are still responsive to oxidative stress (170), perhaps reflecting the distinct roles of these repair factors in replication and transcription. Both NEIL1 and NEIL2 have been found in mitochondria as well as in the nucleus (171,172). NEIL2 has been identified in association with microtubules but the relevance of this interaction is unknown (173). As with NTHL1, NEIL2 activity can be stimulated by the scaffold protein XRCC1 (102). Intriguingly, NEIL1 transcript is subject to RNA editing by the adenosine deaminase ADAR1, which results in a K→R change in the lesion recognition site. The edited form is less efficient at removing thymine glycol, but more efficient at removing 8-oxoguanine oxidation products (174). However, the biological role of this editing is not yet clear.

#### 8-Oxoguanine–DNA glycosylases

The 8-oxoguanine–DNA glycosylase family (human: OGG1; *S. cerevisiae*: Ogg1) is responsible for excising G oxidation products with intact ring systems including the extremely common 8-oxoguanine and additional modifications, specifically across from C (175–188), and G-derived formamidopyrimidine (176,181,186,188). This family of enzymes possesses an AP β-lyase activity specifically across from a C (63,175). Some weak δ-elimination has also been detected (180) and Ogg1 has a minor dRPase activity, perhaps due to δ-elimination coupled with the Tpp1 3′-phosphatase (52). OGG1 and Ogg1 lyase activity is fairly inefficient compared to their glycosylase activities (182) but OGG1 can be stimulated 5-fold in the presence of APEX1 (183,189,190). OGG1 lyase activity can also be replaced by NEIL1/PNK, which binds abasic sites more strongly than OGG1 (191).

OGG1 is expressed as multiple isoforms resulting from alternative splicing. All isoforms contain a mitochondrial matrix targeting signal (MTS), but only isoform 1a contains a strong nuclear localization signal (NLS) (192). Isoform 1a is primarily localized to nuclei and to the nuclear matrix but has also been detected in small amounts in mitochondria (193,194). All the other isoforms, which differ in the C-terminus, are primarily localized to mitochondria, with isoform 2a forming the majority of the mitochondrial pool, associating with the inner membrane (193,195). As with NEIL2, OGG1 associates with microtubules (196), but the function of this interaction has not been elucidated.

OGG1 expression can be upregulated by treatment with alkylating agents and antioxidants, but not by ROS (190,197,198). OGG1 activity may be stimulated by ribosomal protein S3, which may bring OGG1 and APEX1 to lesions; however, S3 can also bind 8-oxoguanine lesions strongly enough to prevent excision (199,200), which might suggest a role in the nucleolus. OGG1 can also be post-translationally modified. PKC phosphorylates OGG1, though the function of this modification is unknown (194), while ROS-inducible p300-mediated acetylation weakens abasic site binding, thus enhancing APEX1-induced OGG1 activity (201).

#### Uracil–DNA glycosylases

The uracil–DNA glycosylase superfamily is one of the most highly conserved and diverse families of BESIR enzymes (202). While *S. cerevisiae* only has one (Ung1), mammals have four (UNG, SMUG1, TDG, MBD4). Mammalian glyceraldehyde-3-phosphate dehydrogenase (GAPDH) and cyclin O (CCNO) have also been reported to have uracil excision activity (203–205). These activities have not been characterized beyond these initial reports, though a 2015 study reported that while GAPDH binds strongly to abasic sites, no uracil excision activity was observed (206). Note that there was some initial confusion in the literature between CCNO and the nuclear UNG isoform UNG2; CCNO was originally named UDG2, which led some early studies of UNG2 to conflate the two proteins.

Uracil–DNA glycosylase (human: UNG; *S. cerevisiae*: Ung1) is the major enzyme responsible for removing U (resulting from C deamination or misincorporation) (207–215) and oxidized derivatives (e.g. 5-hydroxyuracil, alloxan) (216,217), acting on both double-stranded and single-stranded DNA (207,208). UNG localizes to both the nucleus and mitochondria as separate isoforms from alternate promoters (UNG1: mitochondrial; UNG2: nuclear) (218–222). In contrast, Ung1 is a single isoform that localizes to both compartments (223). UNG1 is the only known human mitochondrial uracil–DNA glycosylase. Both UNG2 and Ung1 are upregulated in S phase (218,224–227). UNG2 is controlled by both cyclin-dependent kinases (228,229) and TP53-dependent phosphatase (230,231) and associates with replication complexes (209,232–234). UNG1 is induced with oxidative stress (235). The expression of both human UNG forms are regulated by several miRNAs (236). With a relatively high *K*_M_ compared to the other uracil–DNA glycosylases (237), UNG may be specialized for rapidly detecting and excising lesions encountered by the replication fork and for quickly correcting U misincorporated by DNA polymerase.

The other uracil–DNA glycosylase family members have specialized roles. SMUG1 is nuclear and recognizes the same substrates as UNG1 and UNG2 (216,238–241) in addition to U and T oxidation products (e.g. 5-hydroxymethyluracil, 5-formyluracil, alloxan) (216,239–242) with some weak activity for exocyclic adducts of C (e.g. 3,*N*^4^-ethenocytosine) (243) and deaminated purines (e.g. oxanine, xanthine) (161,244). SMUG1 overall is less efficient than UNG, but SMUG1 prefers single-stranded DNA 100-fold as compared to double-stranded DNA (238). SMUG1 also has a lower *K*_M_ than UNG, which gives it an advantage at lower substrate concentrations (237). As a result, SMUG1 is more efficient at repairing rare lesions in nonreplicating chromatin (245). Additionally, SMUG1 has novel activity removing 5-hydroxymethyluracil from rRNA in the nucleolus (246), presumably to perform quality control on rRNAs while they are unfolded and most vulnerable to damage.

TDG is a nuclear mismatch-specific glycosylase which excises U and T (247–249), highly oxidized 5MeC derivatives (e.g. 5-formylcytosine, 5-carboxylcytosine) (250–252), oxidized T derivatives (e.g. 5-hydroxymethyluracil, 5-formyluracil, thymine glycol) (250,253,254), deaminated A (hypoxanthine) (250), and exocyclic C derivatives (255–258). All substrates are recognized primarily when across from G. TDG has a strong preference for a 5′-adjacent G, as is found in CpG islands (259), and has been strongly associated with both DNA methyltransferases (260,261) and transcription activation (262–266). Recently, a set of 5MeC oxidases, the TET dioxygenases, have been discovered which specifically oxidize the 5-methyl group, converting the methylated base into a substrate for TDG (16,17). Thus, TDG has been implicated in active DNA demethylation as well as reducing the mutation rate of these critical genetic control regions (267). TDG is expressed inversely with UNG, degraded when cells enter S phase and upregulated in G_2_ (268,269), and is translationally regulated by the miRNA miR-29 (270,271). TDG activity is controlled by p300/CBP-SIRT1 acetylation sites which reduce glycosylase activity, adjacent PKCα phosphorylation sites that block acetylation (269,272,273), and transient sumoylation which stimulates abasic site release (274,275).

MBD4 is a recently discovered nuclear mismatch-specific uracil–DNA glycosylase, which is a fusion of a 5MeC binding domain and a uracil–DNA glycosylase domain (276). MBD4 associates with methylated CpG islands (277) and excises U, T and oxidized T derivatives (e.g. 5-formyluracil, thymine glycol) (253,254,278,279) when opposite G. Many other functions have been associated with MBD4 (280). The glycosylase activity of MBD4 has not been fully characterized, but it may be involved in active demethylation similar to TDG (261,281).

#### MutY family glycosylases

The MutY family is a G mismatch-specific adenine–DNA glycosylase (human: MUTYH; *S. cerevisiae*: not present). A:G mispairs are the result of a misinsertion of A across from 8-oxoguanine. MUTYH can excise both A and oxidized A (e.g. 2-hydroxyadenine) across from either G or 8-oxoguanine (282–284). MUTYH is present in both nuclei and mitochondria (193,285) and is upregulated in S phase (286). In the nucleus, MUTYH is associated with replication complexes (286,287) so that mispairs can be corrected shortly after replication. There are multiple known isoforms of MUTYH, but their distinct roles are not known (283,285). MUTYH activity is enhanced by the mismatch repair complex MSH2/6 (288) and APEX1 (289), but it also inhibits OGG1 excision across from, and APEX1 excision of, its resulting abasic site (290). As with NTHL1 and Ntg2, MUTYH also contains an iron-sulfur center, and may be regulated by a similar charge transport mechanism (140).

#### AP endonucleases

AP endonucleases cleave the DNA backbone 5′ to an abasic site or 3′-dRP left behind by AP β-lyase (291–299). AP endonucleases can also recognize oxidized abasic sites (66,67,300). APEX1 and Apn1 have an additional functionality in NIR by directly incising 5′ to oxidized and alkylated pyrimidines (71–77) and they can remove a 3′-terminal lesion with weak exonuclease activity (299,301–304). APEX1 can also cleave at abasic sites within RNA playing a role in RNA quality control (305–307). However, APEX1 has many other known functions due to the redox-sensitive transcription factor domain (reviewed in (308)). Interestingly, while APEX1, APEX2, and Apn2 belong to the exonuclease III family (309), Apn1 belongs to the endonuclease IV family (310).

APEX1 and APEX2 are both upregulated in S phase (311,312) and during DNA damage (313). APEX1 activity is reduced by SIRT1-removed acetylation (314) and CK2-added phosphorylation (315). APEX1 activity is also stimulated by HSP70 binding (316,317), the RAD9-RAD1-HUS1 checkpoint clamp (318), and XRCC1 (319).

APEX1, APEX2, and Apn1 all localize to both the nucleus and mitochondria (294,320–325) while Apn2 is exclusively nuclear (295,296). APEX2 shows the most predominant mitochondrial localization of these enzymes (235). APEX1 and Apn1 make up the majority of AP endonuclease activity in both nuclei and mitochondria with the weaker activities of APEX2 and Apn2 playing minor, backup, or specialized roles (235,294,297,326).

#### Other end-trimming enzymes

Tyrosyl–DNA phosphodiesterase 1 (human: TDP1; *S. cerevisiae*: Tdp1) directly catalyzes the release of a cross-linked topoisomerase 1 from the end-group phosphate. PNKP/Tpp1 then removes the 3′-phosphate and adds a 5′-phosphate to the break site. Topoisomerase II lesions are removed by TDP2 (*S. cerevisiae*: not present), leaving a 5′-phosphate (68). Aprataxin deadenylase (human: APTX; *S. cerevisiae*: Hnt3) directly removes the 5′-5′ AMP left behind by an aborted ligation (69). TDP1/TDP2/Tdp1 and APTX/Hnt3 have all been found in both the nucleus and mitochondria (327). End-trimming enzymes have been reviewed in depth recently (70).

#### DNA polymerases

In humans, X-family DNA polymerases are responsible for the replacement of the excised nucleotides in the nucleus, primarily polymerase β (POLB) and secondarily polymerase λ (POLL). These polymerases may be tightly coupled to APEX1 processing of abasic sites by forming a complex with the XRCC1 scaffold protein (328). Both human polymerases β and λ were reviewed in detail recently (329,330). While *S. cerevisiae* has a POLB homolog, Pol4, it has not been associated with BESIR; Pol4 seems to be involved with gap-filling in double-strand break repair, an activity also associated with polymerase λ (331). Instead, yeast rely on the major replicative polymerase, polymerase δ (Pol3/Pol31/Pol32), to complete repair (332,333). Both human and yeast mitochondrial BESIR appear to utilize the mitochondrial DNA polymerase γ to complete repair of the mitochondrial genome as no X-family polymerases have been identified with mitochondrial localization (334).

#### DNA ligases

Base excision repair is completed by the action of a DNA ligase. DNA ligase I (human: LIG1, *S. cerevisiae*: CDC9) is critical for the completion of nuclear BESIR; in yeast this enzyme plays a more general role, localizing to both nuclei and mitochondria (335–337). The activity of human LIG1 is complemented by DNA ligase III (LIG3), which localizes to both nuclei and mitochondria and specializes in SN-BESIR (338). Both LIG1 and LIG3 in humans associate with the XRCC1 scaffold protein (335). The role of ligases in DNA repair has been reviewed in depth recently (339,340).

### Endonuclease V-mediated excision repair

Recently, a unique repair pathway initiated by endonuclease V (human: ENDOV; *S. cerevisiae*: not present) was discovered (341) (Figure 2E). ENDOV recognizes exocyclic-deaminated purines and cuts the DNA backbone 3′ to the nucleotide immediately 3′ to the lesion, preferentially in single-stranded regions. Through an unknown combination of nucleases, a short single-strand gap is created in the region of the nick, which is filled by DNA polymerase and sealed by DNA ligase. The discovery and current knowledge regarding this enzyme family is reviewed in (342).

### Nucleotide excision repair

The nucleotide excision repair (NER) pathway corrects bulky chemical base adducts and intrastrand crosslinks such as those produced by UV irradiation which induce helical distortion (Figure 2D). NER can partially compensate for BESIR loss (343), and proteins involved in NER are implicated in the repair of certain BESIR substrates (344–348). NER is active in the nucleus and there is no evidence of mitochondrial NER activity. As there are dozens of copies of the small mitochondrial genome, it may be more efficient to degrade severely damaged mitochondrial DNA molecules and resynthesize new ones than to repair them (reviewed in (349)). NER can be induced generally (global genome repair, GGR) or in response to a stalled RNA polymerase (transcription-coupled repair, TCR). In brief, both pathways involve the dual incision of the strand around the damage, leaving a 24–32 nucleotide gap which is filled by a DNA polymerase and sealed with a ligase (350–353).

## OPEN QUESTIONS AND CHALLENGES

The biochemical mechanisms of eukaryotic DNA base damage repair have been elegantly defined by the work of Tomas Lindahl, Aziz Sancar, Paul Modrich and all those who followed. However, much remains to be elucidated regarding the in vivo regulation of the DNA repair pathways, and the interconnections between them and to the core metabolic pathways of the cell. One critical question is how DNA repair pathways could be regulated by partitioning between the nucleus, cytosol, mitochondria, and plastids; recent studies in *S. cerevisiae* have revealed shifts in localization depending on genotoxic stress conditions termed dynamic compartmentalization (133,134). Another critical issue is to understand how lesions are directed into the multiple, redundant pathways available to them; for example, the choice of incision vs. excision in BESIR, endonuclease or lyase processing of abasic sites, or between single-nucleotide and long-patch BESIR. In addition, novel pathways initiated by endonuclease V and RNase H2 will require focused study to determine how they fit in with the established DNA repair landscape.

A major challenge lies in understanding how cells signal the presence of unrepaired base damage, though some pieces of the puzzle are starting to be identified. Recent work has demonstrated that reactive oxygen species are generated in response to DNA base damage in *S. cerevisiae* (354), but the biochemical pathway leading to generation of ROS has not yet been defined. Human OGG1 can allosterically recognize free 8-oxoguanine, one of its major reaction products, which counterintuitively leads to activating cellular signaling pathways including those mediated by the Ras, Rac and Rho small GTPases (355–357). Poly(ADP-ribose) is generated at single-strand breaks, including those produced during BESIR, and may protect the break and recruit repair proteins (358). Several recent studies have shown that an NER-generated single-strand gap can be expanded by exonuclease 1, producing an extended gap that activates the ataxia telangiectasia and Rad3-related (ATR) signaling pathway, connecting NER-repairable lesions to the DNA damage cell cycle checkpoints (359–362). The ability of NER-repairable lesions to activate ATR raises the intriguing possibility that other unrepaired base lesions could activate ATR signaling and checkpoint activation either through NER or through an excision intermediate processed by exonuclease 1. Gaining a clear understanding of these putative signaling pathways and their downstream effectors will be an important advance in elucidating the overall regulation of DNA repair.

Along the same lines as base damage signaling pathways, we have an only rudimentary understanding of how base damage repair is regulated at the level of gene expression and/or post-translational modifications. In a number of cases, transcriptional regulation that occurs in response to genomic insult has been documented but whether additional regulatory mechanisms are critical is not known (363). In only a few specific cases has the functional importance of post-translational modifications of DNA repair proteins been defined (364). Research into these questions will be critical to fully understand how cells respond to and deal with potentially deleterious DNA damage.

Another important issue is to determine how DNA repair pathway regulation and deficiencies are related to human health. A few common inherited polymorphisms have been described, including OGG1 S326C (365), which may have subtle impacts on human health. Other rare inherited variants have been reported in NTHL1 and MUTYH with strong associations to colorectal cancer (34,36). Variations in the abundance and localization of BESIR proteins have also been reported in a number of cancers (366–368). The impact of many of these variations on oncogenesis and patient prognosis is not yet clear. Recently, the relationship of metals to inhibition of BESIR enzymes including the NIEL glycosylases has been a topic of investigation, especially as this inhibition may interact with heavy metal exposures and neurological diseases characterized by metal accumulation (369).

A major challenge to studying DNA damage and repair is the lack of precision tools and endpoints. The majority of our current in vivo knowledge of DNA repair pathways relies on genetic manipulations, genotoxic agents, and mutagenic and phenotypic readouts. Genetic manipulations and genotoxic agents shift lesion abundances in broad, nonspecific ways and their effects are scattered randomly throughout the genome. Mutagenic and phenotypic readouts are several levels removed from the lesions, introducing many opportunities for confounding. Ideally, we would want to introduce a defined lesion at specific loci and then be able to observe how those lesions are processed and resolved. Some recently developed tools and methodologies provide steps in this direction. Micro-irradiation is a promising approach, relying on targeted sensitizers and lasers to provide more localized induction of DNA damage (370). One such tool for the induction of targeted oxidative stress is the KillerRed fluorescent protein derivative, which generates singlet oxygen radicals in response to green light (371). KillerRed has been used to target specific genomic regions with oxidative damage allowing the subsequent response to be detected and analyzed (372). Approaches to measure lesions in a more quantitative and specific manner have also been developed. One method releases lesions from genomic samples using glycosylases and then analyzes their frequencies with chromatography/isotope-dilution tandem mass spectrometry (373). Another method called Excision-seq allows the mapping of classes of lesions by releasing damaged bases with a glycosylase from a genomic sample and subjecting the products to massively parallel sequencing (374); other genome-scale techniques have been reviewed in (375). Techniques to analyze the localization of DNA repair proteins have also been developed as localization of DNA repair proteins has emerged as a potentially important level of regulation. One such method is Q-SCAn, which relies on fluorescent marker proteins to quantify the distribution of proteins among subcellular compartments (376). While all of these novel techniques still have their limitations, advances such as these will be important to continue dissecting the details of DNA base damage and repair.

The 2015 Nobel Prize in Chemistry was awarded in recognition of the seminal work that defined the biochemical mechanisms underlying the critical DNA repair pathways. This work spawned a vitally important field of study, which has greatly improved our knowledge of the details and impacts of DNA base damage and repair. However, there are still major challenges in understanding how these pathways are regulated and integrated with one another to ensure genomic integrity.
